# Transduction of γδ T cells with Baboon envelope pseudotyped lentiviral vector encoding chimeric antigen receptors for translational and clinical applications

**DOI:** 10.3389/fimmu.2025.1548630

**Published:** 2025-06-06

**Authors:** Lorraine Pinot, Aylin Saßor, Nina Möker, Congcong Zhang, Els Verhoeyen, José Villacorta Hidalgo, Rimas J. Orentas

**Affiliations:** ^1^ Research and Development Immunotherapy, Miltenyi Biotec, Bergisch Gladbach, Germany; ^2^ Department of Immunology, Eberhard Karls Universität Tübingen, Tübingen, Germany; ^3^ Faculty of Medicine, Heinrich Heine University of Düsseldorf, Düsseldorf, Germany; ^4^ INSERM U1111, Université de Lyon, Lyon, France and INSERM U1065, Nice, France; ^5^ Université Côte d’Azur, INSERM, C3M, Nice, France

**Keywords:** γδ T cells, chimeric antigen receptor, CAR gd T cells, lentiviral transduction, immunotherapy, allogeneic

## Abstract

γδ T cells represent a promising cell platform for adoptive cell therapy. Their natural anti-tumor reactivity and HLA-independent target cell recognition make them an attractive platform for allogeneic adoptive immunotherapy clinical interventions. Initial clinical trials exploring allogeneic γδ T-cell therapies have demonstrated encouraging safety profiles. However, their therapeutic efficacy, especially against solid tumors, remains limited. This highlights the need for further optimization of γδ T cell products to improve anti-tumor potency, such as the increased targeting induced by the expression of a chimeric antigen receptors (CAR). However, a critical challenge in the development of CAR-γδ T cell therapies has been optimizing transduction efficiency with standard vector formats allowing for optimal CAR transgene expression that then produces an optimal therapeutic product. Here we present an effective method for enhancing CAR transgene expression in γδ T cells using a Baboon-pseudotyped lentiviral vector (BaEV-LV), comparing it to the conventional vesicular-stomatitis-virus-G protein (VSV-G) LVs. BaEV-LV significantly enhanced the transduction efficiency of γδ T cells with CARs, while conserving the beneficial cell product composition and phenotype of untransduced γδ T cells. The γδ T cells transduced with BaEV-LV CARs demonstrated significantly enhanced cytotoxicity against B7H3-expressing tumor cells in both 2D and 3D *in vitro* models. Our findings represent a significant advancement in CAR-γδ T cell engineering, offering a promising new avenue for cancer immunotherapy that combines the unique properties of Vγ9Vδ2 T cells with the targeted specificity of CAR technology. This method is compatible with automated closed-system platforms such as the CliniMACS Prodigy^®^, facilitating Good Manufacturing Practice (GMP)-compliant production for clinical trials. This feature significantly enhances the translational potential of engineered γδ T cells, paving the way for the development of next-generation γδ T cell-based immunotherapies.

## 1 Introduction

The unique biology of gamma delta T lymphocytes (γδ T cells) positions them as a highly promising platform for immunotherapy. Although they constitute only 1-10% of T cells in peripheral blood, their distinctive functional capabilities differentiate them from conventional alpha beta (αβ) T cells, offering exciting potential for innovative therapeutic strategies for cancer and other intractable diseases (1, 2).

One of the most significant advantages of γδ T cells is their ability to recognize a diverse array of antigens without the need for major histocompatibility complex (MHC)-based antigen presentation, allowing their use in allogeneic medical interventions (3). Vγ9Vδ2 T cells, which are the dominant γδ T cell population in peripheral blood (4), react to nonpeptidic molecules called phospho-antigens (pAgs), such as isopentenyl pyrophosphate (IPP) and hydroxymethyl.but-2-enyl pyrophosphate (HMBPP) (5). IPP is an intermediate in the isoprenoid synthesis pathway, which weakly activates Vγ9Vδ2 at physiological conditions, but it accumulates in cancer and infected cells (6). HMBPP is an intermediate in the non-mevalonate pathway present in diverse pathogens, including *Mycobacterium tuberculosis* or *Toxoplasma gondii*. HMBPP is a 10000-fold more potent antigen for Vγ9Vδ2 T cells than IPP (7).

Initial clinical results have also demonstrated significant potential for γδ T cells as a cell therapy platform, owing to their diverse cytotoxic activities against various tumor types and their remarkable ability to infiltrate solid tumors (8, 9). A phase I trial in lung and liver cancers using multiple allogeneic γδ T cell infusions, showed good safety profiles and improved survival (10). More recently, a pilot study of donor-derived ex-vivo expanded Vγ9Vδ2 T cells in patients with high-risk leukemia after haploidentical stem cell transplantation (HSCT), reported safety and relapse free survival (RFS) ≥ 12 months, in contrast to a reported 50% relapse rates at 1 year (11).

Zoledronate, a potent third-generation amino-bisphosphonate used to inhibit bone resorption and to treat bone metastasis and multiple myeloma, is widely employed for the selective expansion and enrichment of Vγ9Vδ2 T cells (12). It inhibits the farnesyl pyrophosphate synthase enzyme which metabolizes IPP, leading to increased intracellular IPP levels in monocytes (13). Its availability in a pharmacy-grade formulation (Zometa^®^) has advanced the application of ex vivo expanded γδ T cells in cancer immunotherapy. Nevertheless, the effective utilization of genetically engineered γδ T cells in solid tumors is contingent upon optimizing their transduction processes. Current clinical trials indicate that untransduced γδ T cells exhibit only moderate efficacy, highlighting the critical need for improved transduction strategies to enhance their therapeutic potential by means of expression of immunotherapeutic payloads, such as tumor antigen-targeted chimeric antigen receptors (CAR) (14).

B7H3 (CD276) has emerged as a promising target for CAR T cell therapy in the treatment of solid tumors. CD276 mRNA is present in the majority of normal tissues; however, the expression of CD276 protein is significantly restricted. This discrepancy is attributed to post-transcriptional regulation by microRNAs (miRNAs) (15). CD276 is frequently overexpressed in numerous solid tumors, notably in breast cancer and brain tumors (16–20), where it contributes to the tumor progression. It is involved in tumorigenesis, the dysregulation of glycolysis and apoptosis, tumor metastasis, and tumor micro-environment (TME) support (21). Because of all these functions, its expression is associated with a poor prognosis (20). There are currently several B7H3 CAR αβ T cells being tested in clinical trials. They have so far shown a good safety profile but a limited efficacy due to tumor resistance mechanisms (22–24).

Lentiviral vectors (LVs) are the primary means to manufacture CAR T cells since they can infect both dividing and non-dividing cells and integrate into the target cell genome, leading to a stable expression of the CAR. Given their extensive development and testing, LVs from third-generation (SIN) vectors are considered relatively safe (25, 26). An advantage of the LV system is the ability to test alternate envelope glycoproteins, i.e. different pseudotypes, to create optimal gene vectors for various target cell populations. Vesicular-stomatitis-virus-G protein (VSV-G) is the most frequently used viral envelope (ENV) for the pseudotyping of lentiviral vectors. It enters mammalian cells through the low-density lipoprotein receptor (LDL-R) that regulates cholesterol metabolism in mammalian cells (27), which explains its limited tropism for unstimulated peripheral blood cells, which express a very low level of LDL-R (28).

While being especially useful for αβ T cell transduction, VSV-G-based LV require a high multiplicity of infection (MOI) and more complex protocols to efficiently transduce γδ T cells (29). This difficulty in using VSV-G based vectors impacts the scalability and practicality of γδ CAR T cell manufacturing processes (30–32). Alternative pseudotypes may provide a key mechanism to overcome this bottleneck. A commonly tested solution is to use retroviral vectors pseudotyped with the RD114 ENV for γδ T cell transduction (33–35). Unlike VSV-G viruses which bind to the target cells via low-density lipoprotein receptor (LDLR), RD114 viruses enter the cells through the amino acid transporter ASCT2. Building on the success of the RD114 pseudotype, another virus from the same family as RD114, the Baboon endogenous retrovirus (BaEV) has been used to transduce a range of cells such as hematopoietic stem cells (HSC) (36), B cells (37), T cells (38) and natural killer (NK) cells (39, 40). Like the RD114 virus, it recognizes the receptor ASCT2, and also recognizes ASCT1, which confers to it a broader tropism (41, 42). Laboratory studies have demonstrated that the BaEV ENV can be successfully used to pseudotype LVs, however prior to this study, their efficacy for transducing γδ T cells has not been reported.

Here we report an optimized and highly effective method for enhancing CAR transgene expression in γδ T cells using the BaEV envelope protein to pseudotype LVs, in direct comparison to the conventional LV ENV, VSV-G. We utilized a third-generation B7H3 CAR for proof-of-concept, testing its efficacy on the B7H3 expressing cell lines MCF-7, MDA-MB-468, and U87-MG.

## 2 Materials and methods

### 2.1 Antibodies and reagents

The following antibodies were used for cell surface staining: anti-human CD107a REA803, anti-human CD14 REA599, anti-human CD19 REA675, anti-human CD27 REA499, anti-human CD3 REA613, anti-human CD45RA REA562, anti-human CD56 REA196, anti-human CD69 REA824, anti-human His GG11-8F3.5.1, anti-human HLA/DR REA805, anti-human KIR2D REA1042, anti-human PD-1 REA1165, anti-human REA Control (S) REA293, anti-human TCR γδ REA591, anti-human TIGIT REA1004 (all from Miltenyi Biotec). The His-tagged B7H3 protein (ACRO Biosystems) was used to assess CAR expression. PBS/EDTA/BSA (PEB) buffer was prepared by adding 0.5% human serum albumin (HAS) (Octapharma) in CliniMACS buffer (Miltenyi Biotec).

### 2.2 Tumor cell lines and spheroids formation

The solid tumor cell lines MDA-MB-468, MCF-7, and U87-MG expressing GFP and Luciferase were used in this study. The wild-type MDA-MB-468 and MCF-7 were obtained from the Leibniz Institute DMSZ and were then transduced with a GFP-P2A-Luc plasmid using a VSV-G LV. U87-MG expressing GFP and Luciferase was kindly provided by Dr. Marius Döring, Miltenyi Biotec. MDA-MB-468, MCF-7, and U87-MG all naturally express B7H3 (**Supplementary Figure S1A**). A modified version of U87-MG in which the target B7H3 had been knocked out with the Alt-R™ CRISPR-Cas9 System (IDT) was used as a control. MDA-MB-468 and U87-MG cell lines were cultured in Dulbecco’s Modified Eagle Medium (DMEM) supplemented with glutamine and sodium pyruvate (Biowest), 10% fetal bovine serum (FBS) (Biowest), and 100mM MEM non-essential amino acids (Gibco). MCF-7 was cultivated in Roswell Park Memorial Institute (RPMI) 1640 medium supplemented with 10% fetal bovine serum (FBS) (Biowest). The cells were maintained in a humidified incubator at 37°C with 5% CO_2_.

To obtain tumor spheroids, U87-MG WT and B7-H3 KO cells were incubated for 4 days in an ultra-low attachment plate (Corning) at 37°C, 5% CO_2_.

### 2.3 LV production

Three different CARs were tested. The B7-H3 CAR is a third-generation CAR with a CD8 transmembrane domain, two co-stimulatory domains derived from CD28 and 4-1BB, and a CD3ζ signaling domain. Previously reported CD19 and CD33 CARs had a CD8 transmembrane domain, a 4-1BB co-stimulatory domain, and a CD3ζ signaling domain (43, 44).

Lentiviral vector (LV) particles were manufactured in suspension HEK293T cells. HEK293T cells were cultivated in Dynamis medium (Gibco) supplemented with Glutamax (Gibco). Cells were transfected with a four-plasmid system using polyethyleneimine (PEI, Polysciences). To produce VSV-G LVs, the ratios of the different plasmids to the 3^rd^ generation transfer vector were 1:0.4 for the envelope, 1:0.3 for the reverse transcriptase and 1:0.6 for gag/pol. For BaEV LVs, it was 1:4 for the envelope, 1:0.1 for the reverse transcriptase and 1:0.2 for gag/pol. After 24h of incubation on an orbital shaker at 165 rpm, 37°C, 5% CO_2_, 5mM sodium butyrate was added (Thermo Scientific). Supernatant was collected 24h later and sterile filtered to remove debris. The LV particles were concentrated via centrifugation (4000 rpm at 4°C for 24h), the pellet was resuspended in TexMACS medium (Miltenyi Biotec) and stored at -80°C. LV particles were titrated after one freeze-thaw cycle on SupT1 cells seeded in serum-free RPMI medium (Gibco), serially diluted and, for the BaEV-LV particles, incubated with Vectofusin-1 (Miltenyi Biotec) for 7 min. Subsequently, LV particles were added onto cultured cells and incubated for at least 96h before being analyzed by flow cytometry. LV titers were calculated by the ratio of transduced cells and LV volume used (TU/ml) (45). The titers are shown in **Supplementary Figure S2** and are in range with the titers reported by Girard-Gagnepain et al. (46).

### 2.4 γδ T cell expansion and transduction

Peripheral blood mononuclear cells (PBMC) were isolated from healthy donors using density gradient separation with Pancoll (PAN-Biotech) according to the manufacturer’s instructions. αβ T cells were then depleted by magnetic separation. Briefly, PBMCs were resuspended in PEB buffer and incubated with anti-human TCR αβ-biotin antibodies (Miltenyi Biotec) for 30 minutes at room temperature. The cells were washed twice and then incubated with anti-biotin microbeads (Miltenyi Biotec) for an additional 30 minutes at room temperature. The cells were washed once more and resuspended in PEB before being separated with a LS column (Miltenyi Biotec). The flow-through, depleted of αβ T cells, was collected and plated at 2 x 10^6^ cells/ml in TexMACS medium (Miltenyi Biotec). The medium was supplemented with 100 IU/ml interleukin-2 (IL-2) (Miltenyi Biotec), 100 IU/ml interleukin-15 (IL-15) (Miltenyi Biotec), and 5µM Zoledronate (Roche).

After 3 days of expansion, cells were transduced with a third generation B7H3 CAR, with either a VSV-G-pseudotyped or a BaEV-pseudotyped LV at an MOI of 1. The BaEV-pseudotyped vector was first incubated with 40µg/L Vectofusin-1 (VF-1) (Miltenyi Biotec) before being added to the cells to enhance transduction efficiency. VSV-G pseudotyped viruses do not benefit from VF-1 for γδ T cells and were thus directly added to the cells (**Supplementary Figure S3**). The cells were further cultivated in TexMACS medium containing 5% human AB serum (Access Biologicals) from day 6 and on. The cultures were maintained in a humidified incubator at 37°C with 5% CO_2_. Medium and cytokines were refreshed every 2–3 days to support cell growth and expansion. The cells were kept in culture for 14 to 15 days before functional analysis, thus 10–11 days after their transduction.

### 2.5 Phenotyping and cell surface markers analysis

Cells were harvested and washed with PEB buffer for phenotyping and cell surface marker analysis. The cells were first incubated with B7H3-His protein (AcroBiosystems) and washed twice. They were then stained for 10 minutes at 4°C with surface marker-specific and anti-His antibodies to assess cellular composition, γδ T cell phenotype (based on CD45RA and CD27 expression), transduction efficiency, and expression of activating and inhibitory markers. 7-AAD (Miltenyi Biotec) was added to exclude dead cells. FCR-blocking reagent (Miltenyi Biotec) was used to decrease non-specific binding. Labeled cells were then washed twice with PEB and read on a MACSQuant 10 flow cytometer (Miltenyi Biotec). The data were analyzed with the FlowJo software version 10.8 (BD Bioscience).

### 2.6 *In vitro* assays for γδ T cell cytotoxicity

γδ T cell function was assessed in 2D models by luciferase-based viability analysis of target cells, on the Incucyte system (Sartorius), and by quantifying their degranulation. γδ T cells were co-cultured with luciferase-expressing target cells (MCF-7, MDA-MB-468, U87-MG WT, and B7H3 KO) at various effector to target (E:T) ratios. After 24h of co-culture, the number of viable target cells was determined by quantifying luciferase expression using the One-Glo luciferase assay kit (Promega) and a Victor 3 plate reader (Perkin Elmer). The Incucyte system was used to monitor cytolysis kinetics over four days by measuring GFP+ target cells every two hours. Finally, to assess the degranulation capacity of γδ T cells, expression of CD107a was measured after two hours of co-culture in presence of CD107a antibody or an isotype control and Bafilomycin A1 (Sigma). The cells were stained with 7AAD, CD3, and anti-TCRγδ antibodies and analyzed using a MACSQuant 10 flow cytometer.

To assess γδ T cell cytotoxicity in 3D models, cells were co-cultured with tumor targets for 6 days in the Incucyte system with GFP+ spheroids of U87-MG WT and B7-H3 KO at different E:T ratios. The total integrated intensity of GFP was measured every two hours.

### 2.7 Statistical analysis

The data were analyzed with GraphPad Prism version 10.1.2 for Windows. Unless stated otherwise, Tukey’s multiple comparisons test with paired data was used. A p-value below 0.05 was considered significant.

## 3 Results

### 3.1 γδ T cells engineered with BaEV-pseudotyped LV successfully express CARs

**Figure 1:**
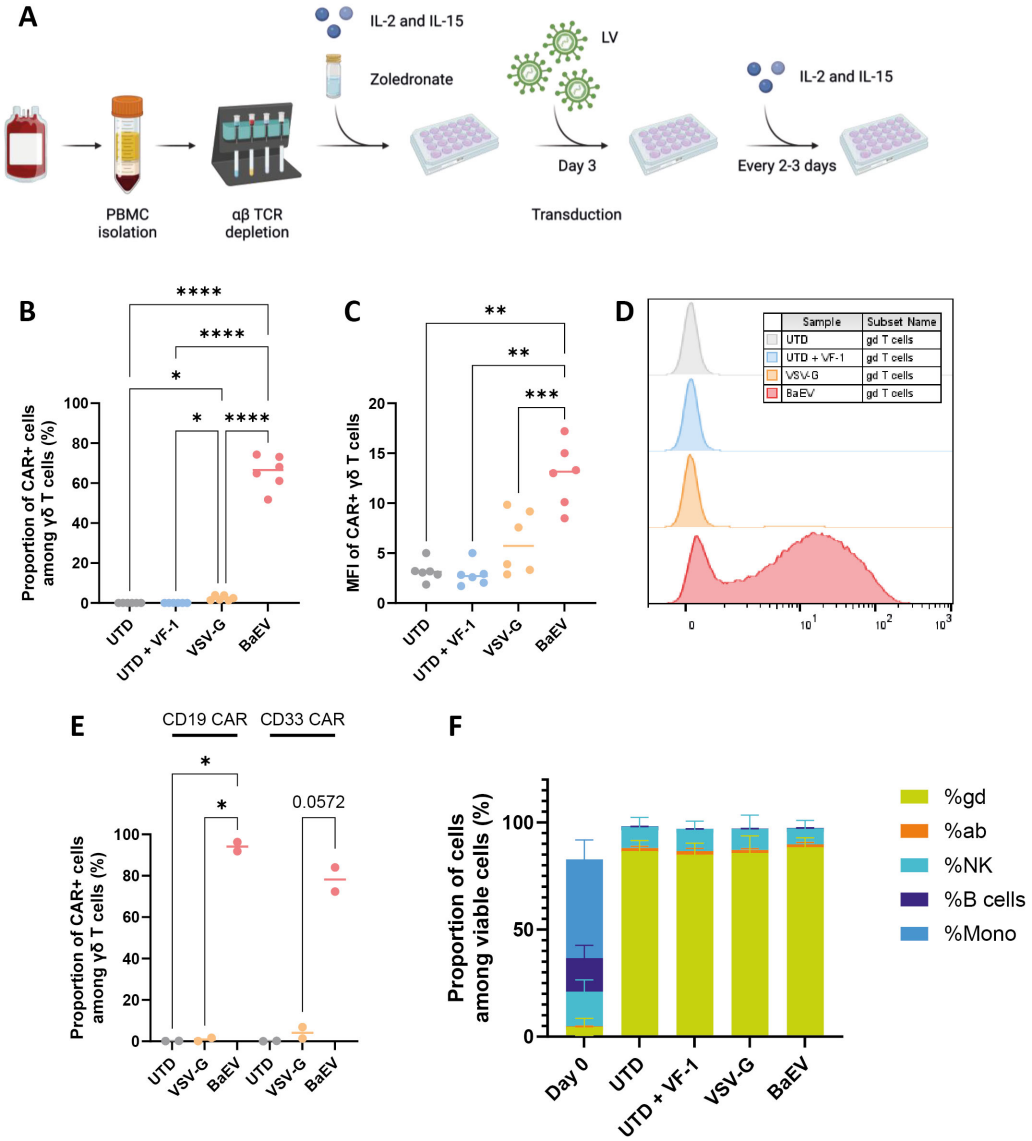
γδ T-cell CAR transduction. **(A)** After three days of expansion, cells were transduced with BaEV or VSV-G pseudotyped LV encoding a B7H3 CAR. VF-1 was used in UTD and BaEV samples. Transduction efficiency **(B)** and the CAR MFI **(C, D)** were measured by flow cytometry at the end of culture. **(E)** After 3 days of expansion, cells were transduced with BaEV-pseudotyped LV encoding either a CD19 or a CD33 CAR and VF-1. Transduction efficiency was measured by flow cytometry at the end of the culture. **(F)** The final cellular product was also analyzed with flow cytometry to determine its cell composition. ns = non-significant, * = p<0.05, ** = p<=0.01, *** = p<=.001 and **** = p<0.0001. Each data point is an individual donor.

γδ T cells were transduced after three days of culture with a third-generation B7H3 CAR LV pseudotyped with either a VSV-G or BaEV envelope (**Figure 1A**). VF-1 was used to enhance transduction efficiency for the BaEV-pseudotyped LV. At a MOI of 1, 65.6 ± 8.4% of γδ T cells expressed the CAR with the BaEV-pseudotyped LV, a significantly higher rate than with the VSV-G ENV (**Figure 1B**). B7H3 CAR surface expression was nearly doubled in γδ T cells transduced with the BaEV-LV (**Figures 1C, D**). This increased transduction efficiency was also confirmed with two additional CAR vectors. A higher proportion of γδ T cells expressed CD19 CAR with BaEV-LVs. A similar trend was observed with CD33 CAR (**Figure 1E**). Transduction did not affect cell product composition, as the final product retained a high proportion of γδ T cells (> 85%), with NK cells as the predominant non-γδ T cells population (**Figure 1F**). These findings demonstrate that the BaEV-LV effectively generates high-purity CAR γδ T cells with robust CAR expression.

### 3.2 Viral transduction does not alter the phenotype of γδ T cells

**Figure 2:**
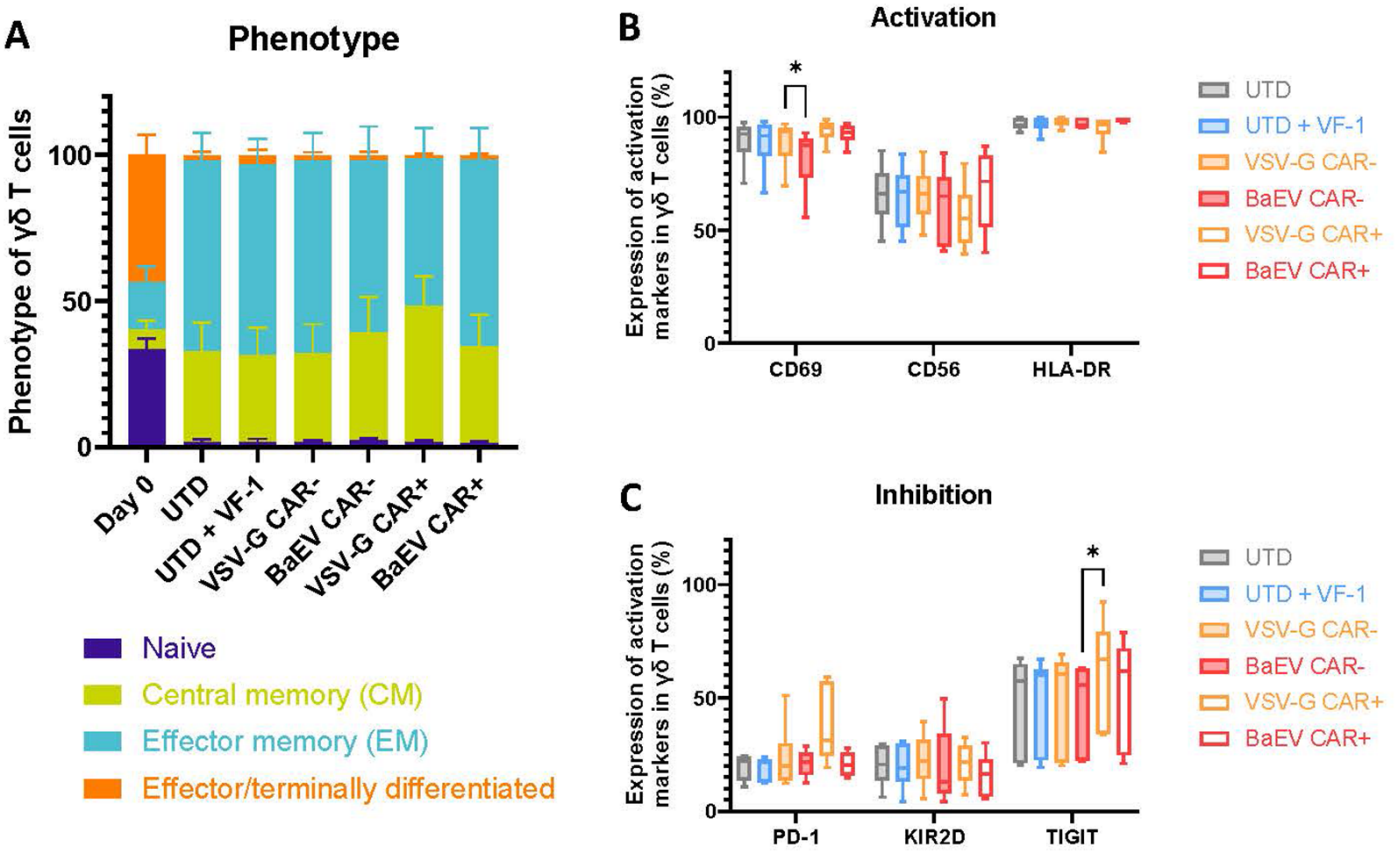
γδ CAR- T cell phenotype and activation profile. **(A)** γδ T cells were analyzed by flow cytometry at the end of culture to determine phenotype based on expression of CD45RA and CD27. **(B, C)** The expression of activation **(B)** and inhibition **(C)** markers by γδ T cells was analyzed by flow cytometry after expansion. Populations analyzed: starting population (Day 0), untransduced γδ T cells (UTD), untransduced γδ T cells supplemented with VF-1 (+VF-1), CAR+ γδ T cells transduced by either BaEV-LV (BaEV CAR+) or VSV-G (VSV-G CAR+) and CAR- γδ T cells in samples transduced by either BaEV-LV (BaEV CAR-) or VSV-G (VSV-G CAR-). * = p<0.05.

We characterized γδ T cells before and after the cultivation period using CD27 and CD45RA expression to determine their phenotype. After 10–14 days of expansion, the proportion of naïve and effector cells (CD45RA+) drastically decreased, with most cells exhibiting either a central or effector memory phenotype (CD45RA-). The phenotype of both CAR+ and CAR- cells was not significantly impacted by VF-1 or LV transduction (**Figure 2A**). Markers for activation, including CD69, CD56 and HLA-DR, were also assessed (**Figure 2B**). CD69 expression was high in all conditions, with a slight reduction only in CAR-γδ T cells transduced with BaEV-LV compared to VSV-G-LV. There was a notable donor variability in CD56 expression, but no significant differences across conditions. HLA-DR expression was consistently high across all transduction conditions. Immune-inhibitory markers PD-1, KIR2D, and TIGIT were also evaluated (**Figure 2C**). PD-1 and KIR2D expression remained low and consistent across samples. TIGIT expression was donor-dependent and only upregulated in CAR+ γδ T cells transduced with VSV-G-LVs, compared to CAR-γδ T cells transduced with BaEV-LVs. Overall, BaEV-LV transduction did not alter γδ T cell phenotype or impede their activation.

### 3.3 B7-H3 CAR T cells engineered with BaEV-LV show superior killing in various solid tumor models expressing CAR targets

**Figure 3:**
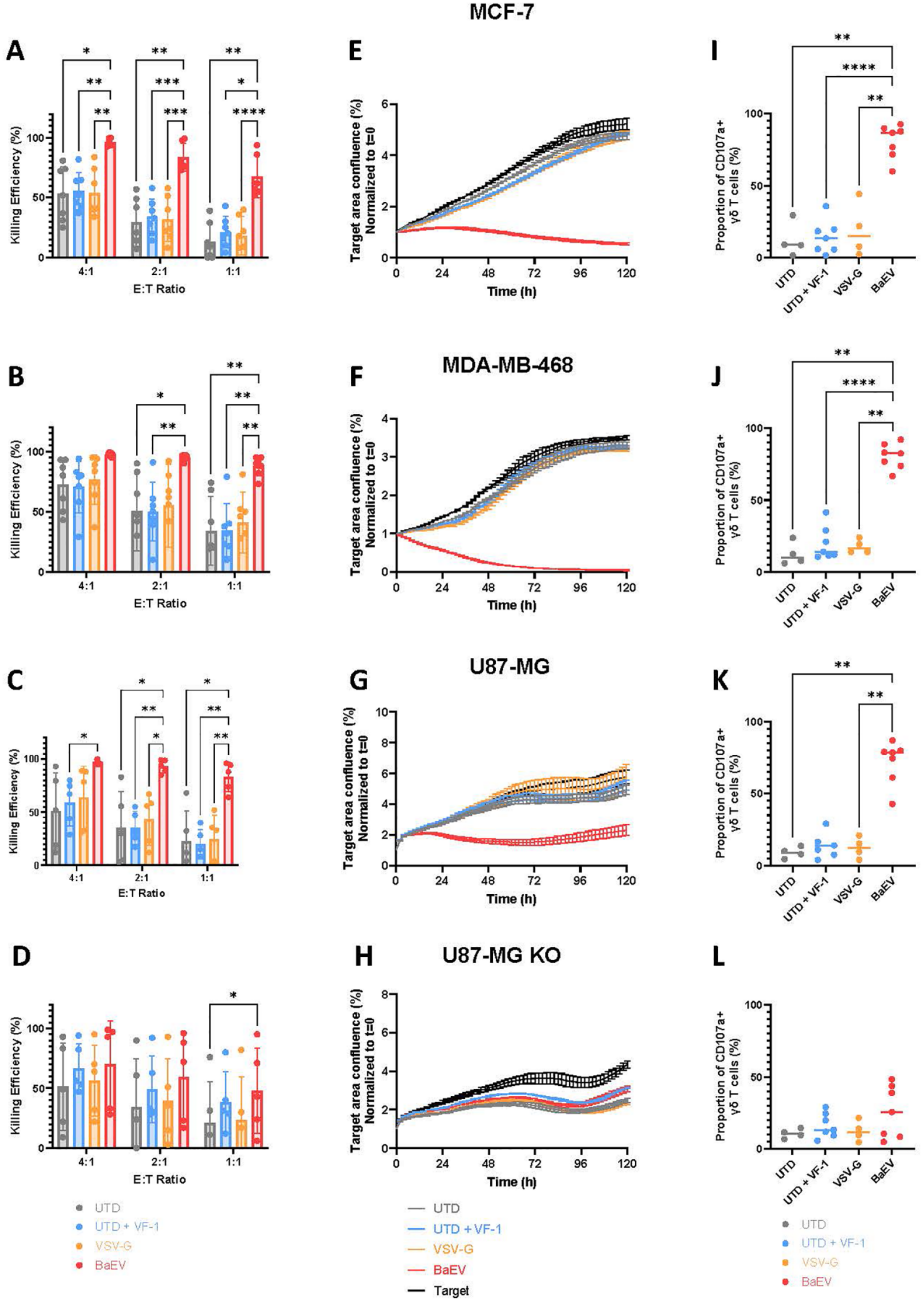
γδ CAR T cell cytotoxicity. B7-H3 CAR γδ T cells were co-cultured with indicated tumor cell lines (ea. row). **(A-D)** Overnight viability of luciferase expressing lines MCF-7 (**A**, n=6), MDA-MB-468 (**B**, n≥6), U87-MG (**C**, n=6) and U87-MG B7-H3 KO (**D**, n≥3) was measured after 24h of coculture with UTD and B7-H3 CAR γδ T cells. **(E-H)** Incucyte analysis with and without γδ T cells at a 1:1 E:T ratio, error bars = SEM. **(I-L)** The expression of CD107as measured by flow cytometry after 2h of co-culture. * = p<0.05 and ** = p<=0.01. Each data point is an individual donor.

To evaluate the cytotoxicity of B7-H3 CAR γδ T cells, we conducted an endpoint killing assay against various B7H3(+) tumor lines. In all three target cell lines, MCF-7, MDA-MB-468 and U87-MG, γδ T cells transduced with a BaEV-LV encoding B7H3 CAR had a significantly higher killing efficacy than either untransduced or CAR-γδ T cells transduced with VSV-G LVs, especially at low E:T ratios (**Figures 3A–C**). In U87-MG B7H3 knockout cells, BaEV-CAR γδ T cells demonstrated enhanced killing only at the lowest E:T ratio compared to untransduced cells (**Figure 3D**). We confirmed these results in an Incucyte killing assay, which measures long-term cytotoxicity kinetics. Only BaEV-transduced B7H3 CAR γδ T cells could control tumor cells expressing the CAR target at a 1:1 E:T ratio (**Figures 3E–G**). There was no difference in killing between the different γδ T cells in the assays targeting U87-MG B7H3 KO cells (**Figure 3H**). Similar results were obtained with both CD19 and CD33 CAR γδ T cells (**Supplementary Figure S4**). To further investigate the cytotoxic mechanism, we assessed expression of CD107a as a marker of degranulation after 2 hours of co-culture with target cells. CD107a expression was significantly increased in BaEV CAR-γδ T cells co-cultured with cancer cells expressing B7H3 (**Figures 3I–K**) but not with B7H3-negative cancer cells (**Figure 3L**).

**Figure 4:**
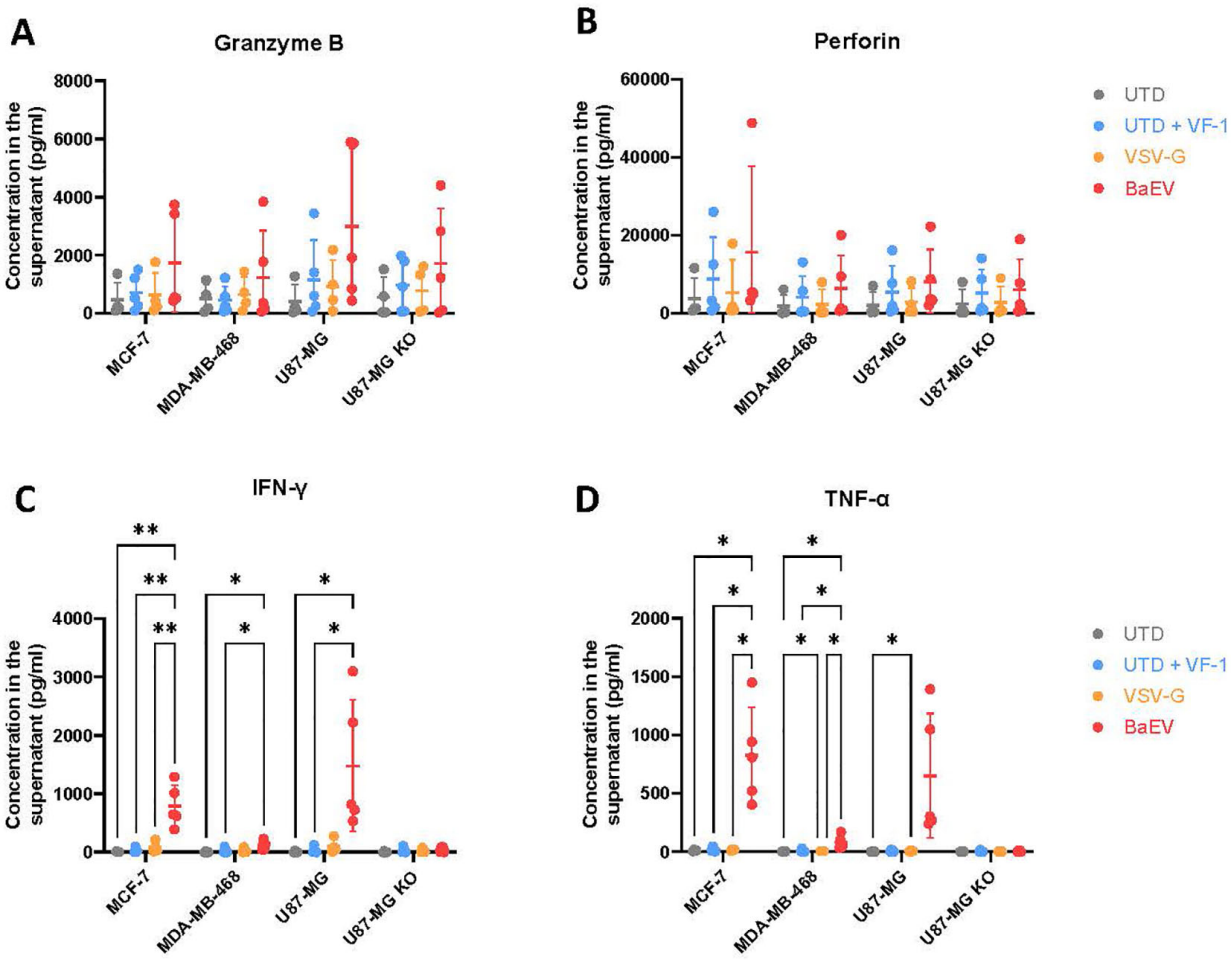
γδ CAR T cell cytokine production. B7-H3 CAR γδ T cells were co-cultured with tumor cell lines (x-axis) for 24h at an E:T ratio of 4:1. Supernatant was collected and analyzed with a T/NK MACSplex kit. Granzyme B **(A)**, perforin **(B)**, IFN-γ **(C)** and TNF-α **(D)** concentrations were detected in samples containing γδ CAR T cells and not in samples with targets alone. n=5. * = p<0.05, ** = p<=0.01. Each data point is an individual donor.

A multiplex cytokine secretion assay revealed no significant increase in granzyme B and perforin secretion, although BaEV-CAR γδ T cells tended to show higher secretion levels (**Figures 4A, B**). Interferon- γ (IFN-γ) and tumor-necrosis factor α (TNF-α) concentrations were significantly elevated in the supernatant of BaEV-CAR γδ T cells co-cultured with cancer cells expressing B7H3 (**Figures 4C, D**). In summary, B7-H3 CAR γδ T cells produced with BaEV-LVs are highly cytotoxic against cancer cells expressing B7H3.

### 3.4 B7-H3 CAR γδ T cells successfully control a U87-MG spheroid model expressing the CAR target

**Figure 5:**
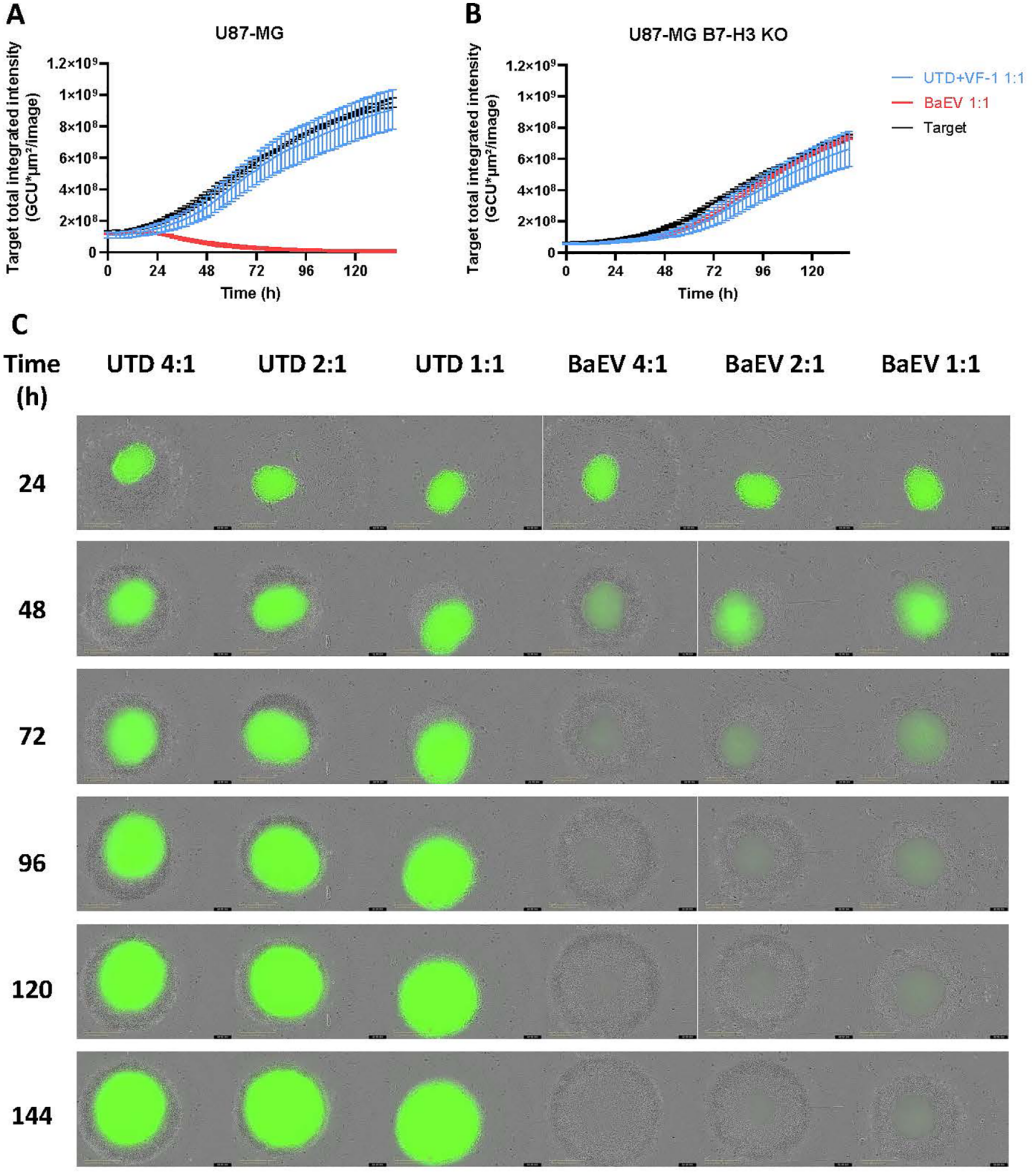
B7-H3 CAR γδ T cells clear U87-MG tumor spheroids. B7-H3 CAR γδ T cells were co-cultured with U87-MG WT or B7H3 KO in an Incucyte device. **(A, B)** GFP integrated total intensity was measured every two hours for both U87-MG **(A)** and U87-MG B7-H3 KO **(B)** with and without γδ T cells at a 1:1 E:T ratio. **(C)** Images for U87-MG GFP expression are shown every 24h for both UTD and B7-H3 CAR γδ T cells co-cultures.

To better evaluate the cytotoxic potential of B7-H3 CAR γδ T cells, we co-cultured them with 3D spheroid models of U87-MG. At a 1:1 E:T ratio, B7-H3 CAR γδ T cells effectively controlled U87-MG spheroids whereas UTD γδ T cells had no effect (**Figure 5A**). In B7H3 KO spheroids, no difference in killing was observed between UTD and CAR γδ T cells (**Figure 5B**). Increasing the E:T ratio led to a dose-dependent increase in B7-H3 CAR γδ T cell-mediated cytotoxicity, which was not observed with untransduced cells (**Figure 5C**).

## 4 Discussion

CAR γδ T cells are a promising alternative modality to treat solid tumors in either autologous or allogeneic interventions. However, there has been a lack of simple and efficient methods to deliver the CAR construct with LVs. This study aimed to evaluate the efficiency of transduction and function of γδ T cells engineered with BaEV-LV to express various CARs, focusing on the transduction efficiency, cellular characteristics, and cytotoxic potential of the engineered cells.

Our results show that BaEV-LVs achieved superior transduction efficiency, with higher CAR expression both in terms of percentage of CAR+ cells and median fluorescence intensity (MFI) compared to VSV-G-pseudotyped LVs. Similar results have been reported in other cell types, such as NK cells (39, 40), B cells (37, 47), and HSCs (36). This high transduction efficiency is facilitated by the compatibility of BaEV-LVs with the soluble peptide VF-1, which provides an advantage over traditional RetroNectin-based methods, especially in the context of scalability for clinical applications (48). We did not observe any difference in terms of growth, phenotype or killing when VF-1 was added to untransduced samples. It has also been reported elsewhere that VF-1 is not toxic for hematopoietic stem cells (49). Importantly, BaEV-LV transduction did not negatively impact the cellular composition, phenotype, or activation/inhibition status of γδ T cells. Our findings indicate that transduced γδ T cells maintained a high level of purity, with most non-T cells being NK cells. NK cells have been reported to have a synergistic action with γδ T cells: their activity is enhanced by γδ T cells (50–52). They have also demonstrated a favorable safety profile in clinical trials so far (53). Their presence is thus advantageous for the safety and efficacy of a γδ T cells-based cellular product. The favorable phenotype observed in the expanded γδ T cell population is advantageous for immunotherapy applications. Memory γδ T cells are associated with greater persistence and potentially enhanced anti-tumor activity upon encountering target cells, an important factor in solid tumor settings where long-term cell activity is essential (54). The activation status of transduced cells was also preserved, without evidence of tonic signaling or excessive activation, which is crucial for preventing premature exhaustion (55, 56). CAR- γδ T cells in the BaEV transduced condition expressed less CD69 than those in the VSV-G transduced condition, which could suggest that cells expressing the highest amount of CD69 were more favorably transduced by the BaEV LV. The stability of inhibitory markers, including PD-1, KIR2D, and TIGIT, suggests that BaEV CAR γδ T cells are not more prone to exhaustion and maintain a robust anti-tumor potential.

BaEV CAR γδ T cells demonstrated high efficiency in selectively killing B7H3-positive tumor cells, particularly against MDA-MB-468, MCF-7, and U87-MG cell lines. The selectivity of BaEV CAR γδ T cells for target-positive cells underscores their therapeutic precision, reducing the likelihood of off-target effects. Mechanistic studies revealed that these cells exhibited increased degranulation (CD107a expression) and secreted higher levels of pro-inflammatory cytokines such as IFN-γ and TNF-α when exposed to B7H3-positive target cells, suggesting that cytotoxic mechanisms are driven in part by both degranulation and cytokine release. This dual mechanism may enhance the durability and potency of γδ T cell-mediated tumor clearance. There may be additional mechanisms available to γδ cells *in vivo* that were not available in the *in vitro* setting of this study. γδ T cells have also been shown to induce antibody-dependent cellular cytotoxicity (ADCC) (57, 58), thus further activating anti-tumor immunity (59–65). The relevance of these findings is supported by our observations in 3D tumor spheroid models. Unlike 2D cell cultures, 3D models better simulate the tumor microenvironment, including hypoxic gradients and physical cell-to-cell interactions (66). BaEV CAR γδ T cells effectively controlled tumor growth at low E:T ratios in 3D spheroids, a promising result for translating these therapies to solid tumors, where T cell infiltration and persistence are often challenging.

Our study demonstrates that BaEV-pseudotyped LVs offer an efficient and scalable means of engineering γδ T cells to express CARs, preserving cell phenotype and activation status while achieving high levels of transduction and cytotoxicity. These BaEV CAR γδ T cells hold significant promise for solid tumor immunotherapy, particularly for targeting B7-H3-positive cancers. These features, combined with the potential for scalable manufacturing, make BaEV LV-generated CAR γδ T cells a promising candidate for clinical development.

## Data Availability

The raw data supporting the conclusions of this article will be made available by the authors, without undue reservation.
