# Supplementary material for: Transduction of γδ T cells with Baboon envelope pseudotyped lentiviral vector encoding chimeric antigen receptors for translational and clinical applications
Source: Front Immunol. 2025 Jun 6;16:1548630. doi: 10.3389/fimmu.2025.1548630 (PMC12179110; doi:10.3389/fimmu.2025.1548630)
Supplement: Supplementary file 1 [file DataSheet1.docx]

Supplementary Material

# Supplementary Figures and Tables


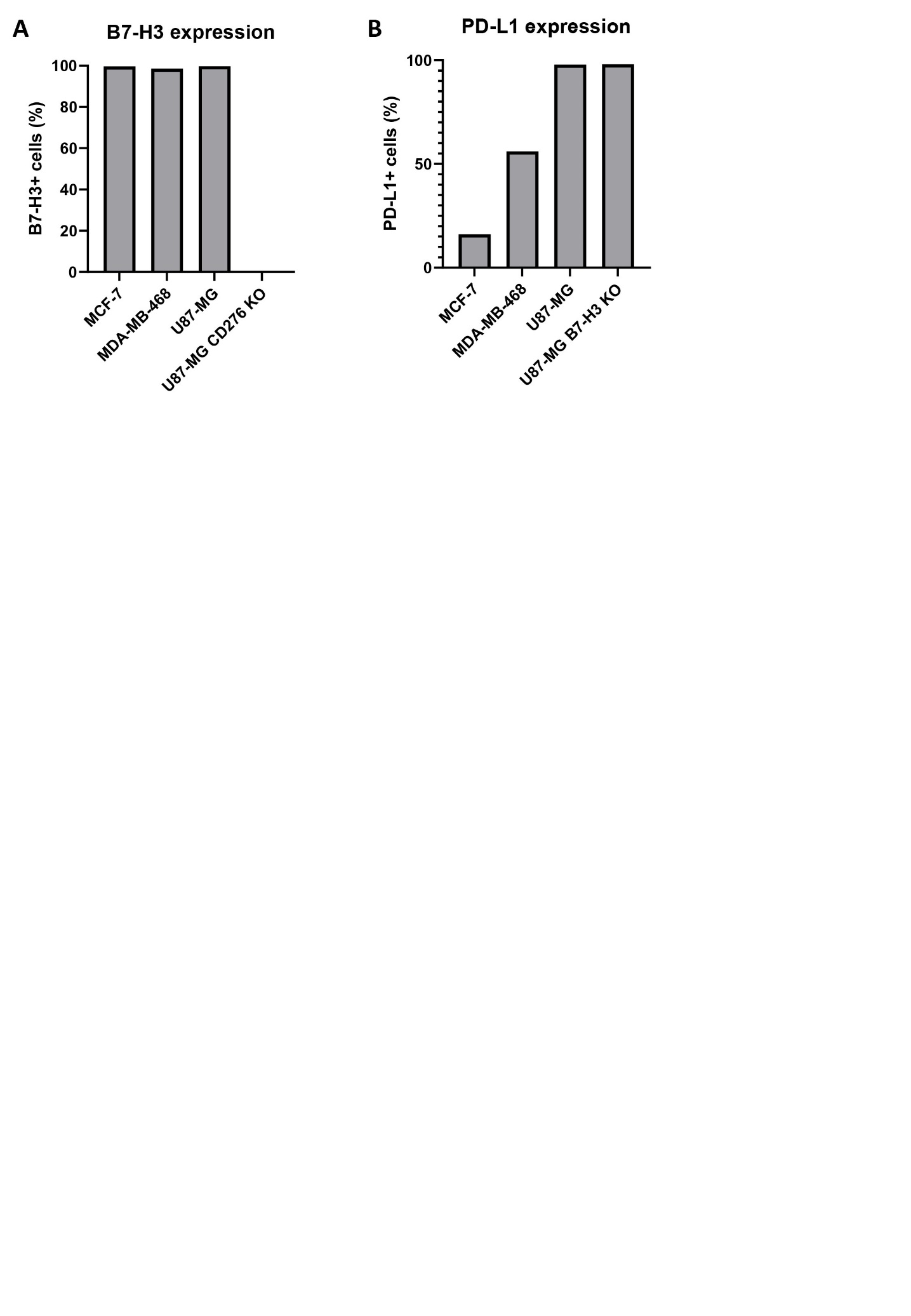


**Supplementary Figure 1.** Expression of B7-H3 (A) and PD-L1 (B) on live cells was measured by flow cytometry.

**Supplementary Figure 2.** Titers of LV particles pseudotyped with either the BaEV or the VSV-G envelop. SupT1 T cells were transduced with serial dilutions of BaEV LVs or VSV-G LVs. The transduction efficiency was measured by flow cytometry and used to calculate the titer in TU/ml.

**Supplementary Figure 3.** The transduction efficiency of γδ T cells with a B7H3 CAR by VSV-G LV in presence or absence of VF-1 was determined by flow cytometry 10 days after transduction.


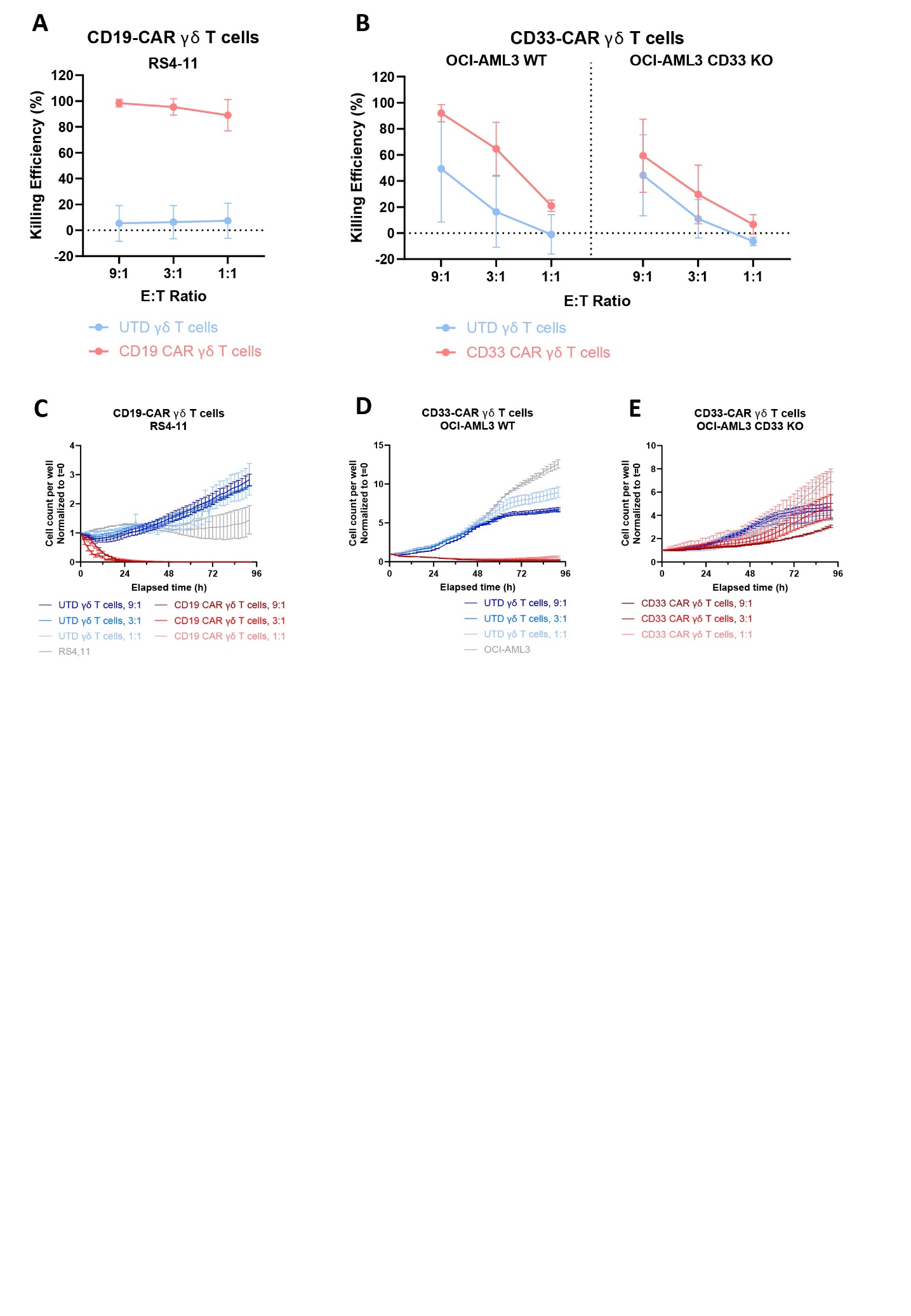


**Supplementary Figure 4.** γδ T cells exhibit increased cytotoxicity upon CAR transduction. Cytolysis mediated by γδ CAR T cells, was analyzed by co-culture with hematological tumor cell lines. (A-B) Expression of luciferase by Luc+ RS4-11 cocultured with UTD and CD19-CAR γδ T cells (A, n=3) and OCI-AML3 WT or CD33 KO cocultured with UTD and CD33 CAR γδ T cells (B) was measured at different E:T ratios. (C-E) The expression of GFP by GFP+ RS4-11 (C), OCI-AML3 (D) and OCI-AML3 CD33 KO (E) was measured in an Incucyte instrument with and without γδ T cells at an 1:1 E:T ratio. Error bars = SEM.
